# Clinicopathological Features and Increased Expression of Toll-Like Receptor 4 of Gastric Cardia Cancer in a High-Risk Chinese Population

**DOI:** 10.1155/2018/7132868

**Published:** 2018-02-18

**Authors:** Guangcan Chen, Muming Xu, Jingyao Chen, Liangli Hong, Wenting Lin, Shukun Zhao, Guohong Zhang, Guo Dan, Shuhui Liu

**Affiliations:** ^1^Department of Gastrointestinal Surgery, The First Affiliated Hospital of Shantou University Medical College, Shantou, Guangdong Province 515041, China; ^2^Department of Abdominal Surgery, The Tumor Hospital of Shantou University Medical College, Shantou, Guangdong Province 515041, China; ^3^Department of Pathology, Shantou University Medical College, Shantou, Guangdong Province 515031, China; ^4^Department of Pathology, The First Affiliated Hospital of Shantou University Medical College, Shantou, Guangdong Province 515041, China

## Abstract

The incidence of gastric cardia cancer (GCC) is high in China. However, the clinicopathological characteristics and the carcinogenesis of GCC are unclear. Toll-like receptor 4 (TLR4) is an important innate immunity receptor and has a role in non-GCC (NGCC). We compared the clinicopathological characteristics of GCC patients from a high-risk area in China to esophageal cancer (EC) patients. Immunohistochemistry for TLR4 was performed in 201 histological samples of normal gastric cardia mucosa (*n* = 11), gastric cardia inflammation (*n* = 87), and GCC (*n* = 103). We included 84 patients with EC and 99 with GCC. GCC tissue was more poorly differentiated than EC tissue and more invasive, with more histomorphologic variation. Lymph node metastasis was more frequent in GCC than in EC. The *Helicobacter pylori* infection rate was higher but not significantly with GCC than EC. Survival was shorter with lymph node metastasis. We found a statistically significant trend for progressive increase of TLR4 expression from normal mucosa to inflammation in GCC. GCC in this high-risk area displays clinicopathologic characteristics different from those of EC and different from those of gastroesophageal junction carcinomas in other countries, although this was not analyzed statistically. Increased TLR4 expression in gastric cardia lesions may be associated with GCC tumorigenesis.

## 1. Introduction

Gastric cancer is the fifth most common malignancy, with an estimated 952,000 new cases in 2012 worldwide [1]. Although gastric cancer is still a major contributor to the global cancer burden, its incidence has decreased over the past decades [1].

Generally, gastric cancers can be classified into two categories: gastric cardia cancer (GCC) arising in the area of the stomach adjacent to the gastroesophageal junction (GEJ) and non-GCC (NGCC) arising from more distal regions of the stomach [2]. The incidence and risk factors for GCC and NGCC vary considerably. The decreasing incidence of gastric cancer is due mostly to the declining trend of NGCC; however, the incidence of GCC may be increasing [3–5]. In addition to different epidemiology, GCC and NGCC are thought to have different risk factors, clinicopathologic features, and gene expression [6–8]. Thus, current research is addressing GCC and NGCC as separate diseases.

From the *Cancer Incidence in Five Continents, Vol. X* (CI5X) and “GLOBOCAN 2012 v1.0, cancer incidence and mortality worldwide: IARC CancerBase No. 11” [9, 10], Colquhoun et al. estimated 260,000 cases of GCC worldwide in 2012, comprising 27% of the total gastric cancer cases, most of which occurred in Eastern/Southeastern Asia (59%), followed by Central Asia (15%). More than half of the total cases occurred in China (135,000; 52%) [2]. In high-risk areas in Asia [11–13], the incidence of GCC shows a characteristic geographic aggregation with esophageal cancer (EC). China has six high-risk EC regions, including the Taihang Mountain area, the Qingling Mountain area, North of Sichuan Province, the Dabie Mountain area, East of Guangdong Province, and the Subei area [5]. The Chaoshan high-risk area east of Guangdong is the only littoral high-risk area. Our previous epidemiological study showed an extremely high incidence of EC (74.47/100,000) and GCC (34.81/100,000) on Nan'ao Island in the Chaoshan area from 1995 to 2004 [11].

EC and GCC used to be treated as a single disease because of the coincidence of the two cancers, similar clinical symptoms, and limited techniques to distinguish them. Since the 1990s, with widespread use of endoscopy and a new classification by the World Health Organization (WHO), GCC is diagnosed as a disease different from EC in high-risk areas in China; however, the tumorigenesis, pathogenesis, development, and prognosis of GCC from high-risk areas are poorly understood. Although GCC has the same geographic distribution as EC in China [14], previous study showed that they differ in histopathology, although this was not analyzed statistically [15]. Recent epidemiological data have shown that the incidence of EC is declining as compared with an increasing trend of GCC in high-risk areas [16]. GCC in high-risk areas is also different from carcinomas of the GEJ studied primarily in Caucasian populations, which are believed to be related to gastroesophageal reflux disease (GERD) [3] and Barrett's esophagus (BE) [17, 18]. BE-related GCC are rare in China [19–21]. GERD prevalence in China was reported in some areas of China, and it is reported GERD rates in Hong Kong, China, have risen over the last decade. However the incidence rate of GERD in China is still much lower than that reported in Western countries [22–24].

*Helicobacter pylori (H*. *pylori)* infection has been confirmed as an important factor for distal gastric cancer [25]. Its relationship with GCC remains controversial [26–29]. Our previous study suggested that persistent *H*. *pylori* infection and the related chronic inflammation may contribute to the high incidence of GCC in the Chaoshan high-risk area [29]. Toll-like receptors are essential for *H*. *pylori* recognition and they initiate inflammatory pathways that may acquire oncogenic potential [30–32]. Toll-like receptor 4 (TLR4) can recognize lipopolysaccharide, a component of the bacterial cell wall [33]. The relationship between TLR4 and gastric cancer has been well studied [34–38]. However, most of these studies did not distinguish GCC from NGCC. No study has addressed TLR4 protein expression restricted to gastric cardia subtype.

In the present study, we compared the clinicopathologic features of GCC and EC in the Chaoshan high-risk area in China. TLR4 expression was evaluated by immunohistochemistry in normal gastric cardia mucosa, chronic gastric cardia inflammation, and GCC, to better understand the potential role of TLR4 in gastric cardia carcinogenesis. Despite different classifications and definitions of cancers originating around the GEJ [39, 40], we defined GCC as carcinoma in which the epicentre is ≤2 cm below the GEJ, the most accepted definition in China [16].

## 2. Results

### 2.1. Clinicopathological Results

We included 84 patients with EC (male : female ratio 3 : 1 and mean age 57.14 ± 10.28 years) and 99 with GCC (male : female ratio 5.6 : 1 and mean age 62.7 ± 7.79 years). Patients with EC were younger than those with GCC. Although more GCC than EC patients were male, the difference in the male : female ratio between the two groups was not considerable (Table 1).

**Figure 1:**
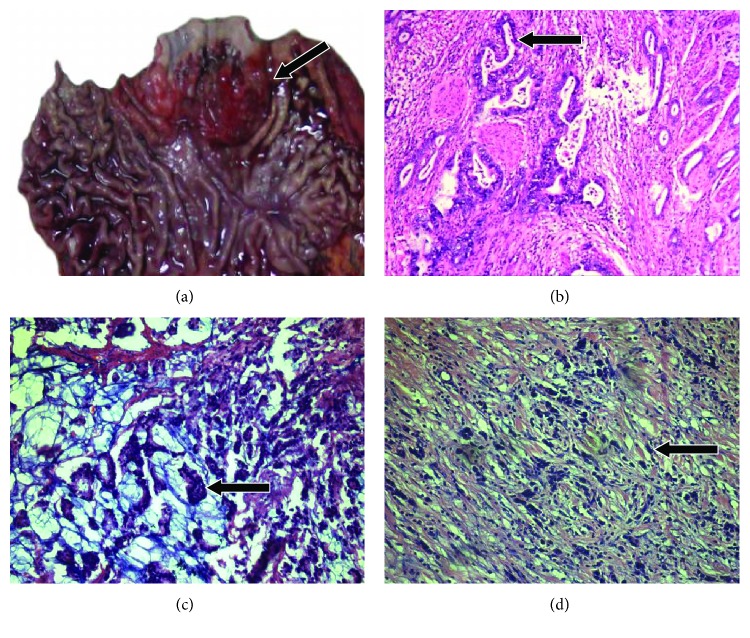
Representative images of gastric cardia cancer (GCC). (a) Gross image of GCC below the gastroesophageal junction (arrow). (b) Histology of GCC with tubular formation (arrow). (c) Histology of mucinous GCC, arrow indicating tumor cells. (d) Histology of small-cell undifferentiated carcinoma, arrow indicating tumor cells.

**Figure 2:**
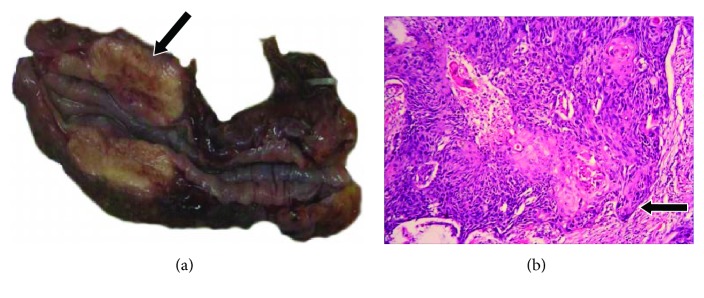
Representative images of esophageal cancer (EC). (a) Gross image of EC (arrow). (b) Histology of squamous cell EC, arrow indicating cancer nest.

The median tumor size was larger with GCC than that with EC (5.92 ± 2.22 versus 4.98 ± 1.51 cm; *P* = 0.001) (Table 1). For 66 EC tumors (78.57%), the epicentres were in the middle thoracic part of the esophagus (Table 1). Representative gross images of EC and GCC are in Figures 1 and 2. The microscopic features of the tumors are summarized in Table 2. Compared with EC tumors, GCC tumors were significantly more poorly differentiated (*P* < 0.001) and exhibited a wider histopathological spectrum. All 84 EC tumors (100%) were squamous cell carcinoma. In contrast, 75.8% of GCC tumors were tubular adenocarcinoma; the remainder were mucinous carcinoma (16.2%), adenosquamous carcinoma (4%), small-cell undifferentiated carcinoma (*n* = 2, 2%), squamous cell carcinoma (*n* = 1, 1%), or a tumor of neuroendocrine phenotype (*n* = 1, 1%). Representative histology images are in Figures 1 and 2.

A significantly higher proportion of patients with GCC than EC showed advanced-stage disease (*P* < 0.001). In addition, a higher proportion of patients with GCC showed lymph node metastasis and deep infiltration (*P* = 0.002 and *P* = 0.009, respectively, Table 2). The tumor tissues from 25/44 patients with EC (56.81%) and 49/83 with GCC (59.03%) were positive for *H*. *pylori* cytotoxin-associated gene A (CagA), a virulence factor that may damage the gastric mucosa and cause inflammation and cell death. Almost all of the East Asian *H*. *pylori* strains are CagA positive. Detecting CagA can be used to detect CagA-positive *H*. *pylori* infection [41]. We found no significant difference in the rate of *H*. *pylori* infection between EC and GCC groups (*P* = 0.59) (Table 3).

### 2.2. Patient Survival

**Figure 3:**
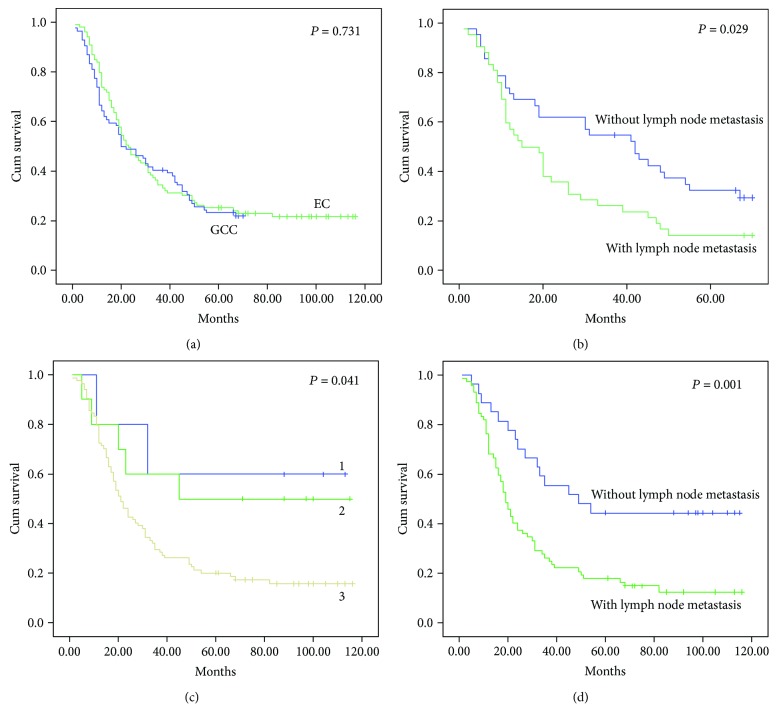
Kaplan-Meier survival curves for patients with GCC and EC. (a) Overall survival. (b) Cumulative survival with EC with and without lymph node metastasis. (c) Cumulative survival with GCC by TNM stage. 1: stage 1, 2: stage 2, 3: stage 3. (d) Cumulative survival with GCC with and without lymph node metastasis.

Survival did not differ among patients who received surgery alone and both surgery and adjuvant therapy, so we chose all 84 EC patients and 99 GCC patients who underwent surgery for survival analysis. The mean survival was shorter with EC than that with GCC (32.14 months, 95% CI 26.796–37.477 versus 43.05 months, 95% CI 34.933–51.165) but not significantly (*P* = 0.731) (Figure 3(a)). The cumulative survival with EC was better without than with lymph node metastasis (38.83 ± 4 versus 25.45 ± 3.4, *P* = 0.029) (Figure 3(b)). The cumulative survival of patients with GCC was better with higher than lower TNM stage (76.4 ± 20.27 and 67.7 ± 15.28 versus 37.86 ± 4.09, *P* = 0.041; Figure 3(c)) and without than with lymph node metastasis (65.67 ± 8.75 versus 34.19 ± 4.17, *P* = 0.001; Figure 3(d)). On multivariable analysis, lymph node metastasis was independently associated with survival with GCC (HR 2.02, 95% confidence interval [CI] 1.058 to 3.837, *P* = 0.033).

The 3-year survival rate was better for patients with EC than that with GCC (40.5% versus 34.3%), but the 5-year survival rate was poorer with EC than that with GCC (22.6% versus 25.3%). Neither of these differences was statistically significant (*P* = 0.392 and *P* = 0.678, resp.).

### 2.3. TLR4 Expression in Gastric Cardia Specimens

**Figure 4:**
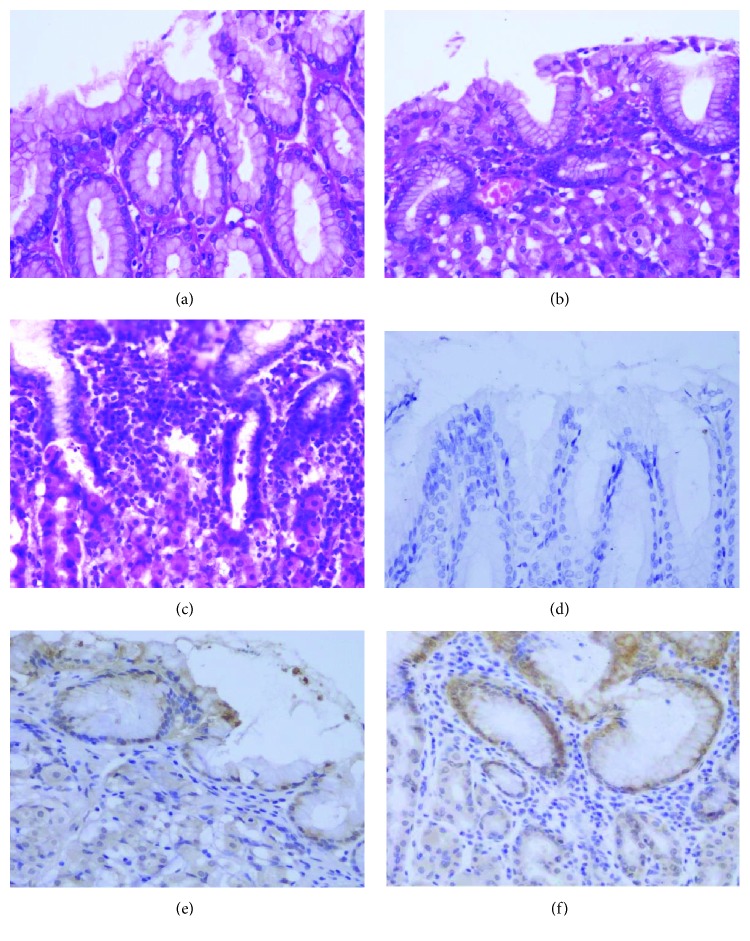
Representative IHC staining for TLR4 in gastric cardia mucosae with different degrees of chronic inflammation: (a) normal, (b) mild inflammation, (c) severe inflammation, (d) no immunostaining in normal mucosae, (e) weak positive staining in mucosae with mild inflammation, and (f) moderate positive staining in mucosae with severe inflammation.

**Figure 5:**
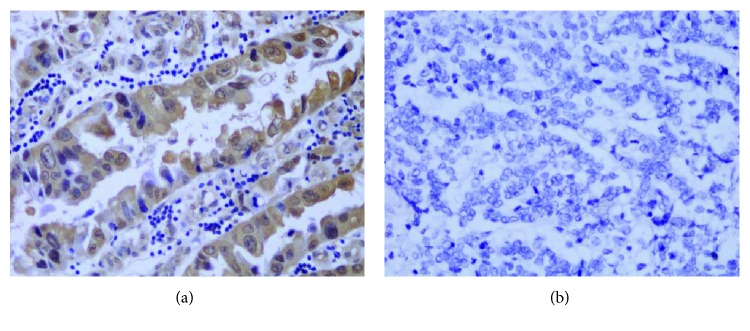
IHC staining for TLR4 in GCC tissue. (a) Strong positive staining in well-differentiated GCC cases with tubular structure. (b) Negative TLR4 staining in poorly differentiated tumor.

In Table 4 and Figures 4 and 5, TLR4 expression in gastric cardia tissue is shown. Among the 98 nonmalignant gastric cardia tissues examined, 11 normal epithelia without inflammation had the lowest score for TLR4 expression, and TLR4 was not detectable in 5 of them. On immunohistochemistry, TLR4 expression was higher in inflamed epithelia than that in gastric cardia mucosa (Figure 4) but did not differ between mild and severe inflammation. TLR4 expression was detectable in most of the 97/103 GCC cases (94.17%). Moreover, strong TLR4 staining was found in well- and moderately differentiated GCC cases with tubular structures but weak or negative TLR4 staining in poorly differentiated tumors (Figure 5). In mucosa with inflammation, TLR4 was expressed in a polarized manner, particularly at the basolateral membrane. In contrast, cancer cells expressed TLR4 diffusely throughout the cytoplasm even in the nucleus. We found a statistically significant trend for increasing TLR4 expression from normal mucosa to gastric cardia inflammation and carcinoma (*P* < 0.05) (Table 4).

## 3. Discussion

In the present study, we compared clinical-pathological features between EC and GCC and described for the first time TLR4 expression restricted to the gastric cardia epithelium. Patients with GCC were significantly older and had a higher male : female ratio than patients with EC, although the difference in the latter variable was not considerable. 92.92% (92/99) of GCC patients were older than 50 years, and 72.62% (61/84) of EC patients were older than 50 years, indicating middle age and elderly people are high-risk group for both GCC and EC, especially for GCC. The mean age for GCC increased compared to the mean age of patients in the 1980s and 1990s [42]. It might be related to the Chinese social aging. A male predominance in the incidence of GCC and EC has been reported worldwide [11, 42, 43], and the male predominance is weaker in EC than that in GCC in this study. An assessment in China high-risk area for GCC indicated male : female ratio ranged from 1.68 to 5.6 [15, 16]. The reason for male predominance in GCC is still unclear. Although tobacco smoking is more prevalent in men than in women in China, the male predominance of GCC is unlikely to relate to this factor. A cohort study following 2 million person-years at risk indicated that the male predominance in GCC was similar among smokers and nonsmokers [44]. In China high-risk area, the male predominance in GCC may be caused by some sex-related genetic factors which need to be further studied.

GCC tumors were, on average, larger and more poorly differentiated, were of higher pathological stage, and were more likely to have lymph node metastasis and deeper invasion than EC tumors. Patients with GCC were more likely to have *H*. *pylori* infection, although the difference did not reach statistical significance. The GCC group had more histological variants than the EC group. GCC included adenocarcinoma, squamous cell carcinoma, adenosquamous carcinoma, small-cell undifferentiated carcinoma, and neuroendocrine carcinoma. In contrast, in ECs, all tumors were squamous epithelial cell carcinoma. The most common location for EC in our sample was the middle thoracic part of the esophagus, whereas in the Western countries, EC originates mainly from the lower part of the esophagus, and the most common histological type is adenocarcinoma [17, 45]. Survival was poorer for both EC and GCC patients with than without lymph node metastasis. We found similar overall survival among our EC and GCC patients, even though the latter had a significantly higher proportion of lymph node metastasis and stage 3 and 4 tumors at the time of diagnosis. In light of these observations, GCC in high-risk areas in China has clinical and pathological features that differ from those of EC from the same area.

Although different studies have shown some shared genetic risk factors between EC and GCC from high-risk areas in China [14, 46–48], the different histology and surrounding anatomical structure of the esophagus and gastric cardia and respective risk factors indicate differences between EC and GCC [49].

Huang et al. performed a study of the clinical and pathological features of GEJ carcinomas in Chinese and US patients. In terms of their data from the United States, patients with GCC in our group showed larger tumor size (5.92 ± 2.22 cm versus 3.5 ± 2.2 cm), lower 3-year (34.3% versus 43%) and 5-year (25.3% versus 28%) survival, and higher disease stage than the US patients [45]. Compared with adenocarcinoma of the GEJ in patients with BE mucosa from America, our patients with GCC showed deeper invasion, higher disease stage, more lymph node metastases, and lower 5-year survival [50]. When comparing data on adenocarcinoma of the GEJ in patients from Japan, with the center located between 1 cm above and 2 cm below the GEJ [51], for our patients, the depth of tumor invasion was deeper, nodal metastases were more frequent, and the differentiation and 5-year survival were worse. In a meta-analysis, the 5-year survival for patients with cancer of the gastric cardia varied from 35% to 54.6% in China [52, 53]. All of these studies reported higher survival rates than we found in our study. Although these studies had discrepancies and overlapping descriptions of tumor location, careful consideration of these results suggests that GCC in the Chaoshan high-risk area in China is more aggressive with a worse prognosis.

Adenocarcinoma of the distal esophagus and Barrett's esophagus-related diseases remain uncommon in China. A large-scale longitudinal clinical and histological data was analyzed on 5401 esophageal cancer (EC) patients diagnosed during 10-year period (2002–2011) at Henan Taihang Mountain high risk area in China. All 217 esophageal adenocarcinoma (EAC) patients from these 5401 EC patients were examined, and EAC was relatively rare and accounted for approximately 5% of all esophageal cancers. Only 10 out of 217 (4.6%) EAC cases were detected to have any evidence of Barrett's esophagus [20]. Though the GERD prevalence in GCC high-risk area in China is unclear, the prevalence of GERD symptoms in South China has varied from 2.3% to 3.8%, much lower than that in the Western countries [22, 23]. Patients with GCC in China and those in the Western countries might have different genetic polymorphisms, lifestyles, diet, and environmental influences [45]. *H*. *pylori* infection is the primary risk factor for distal gastric carcinomas in the Chinese population [54]. A case–cohort study with long-term follow-up in the Linzhou high-risk area found a strong association of GCC with *H*. *pylori* infection [55]. Our previous study found that *H*. *pylori* infection may contribute to the high incidence of GCC and esophageal squamous cell carcinoma in the Chaoshan region [29, 56]. Gastric carcinomas develop on the background of chronic active *H*. *pylori* gastritis via the epithelial precursor lesions. It is believed that a virulent bacterium in a genetically susceptible host is associated with more severe chronic inflammation, and this long-term inflammation may lead to cancer [31, 57]. Observing the adjacent tissue of GCC, we found that most cases showed chronic inflammatory cell infiltration. We speculated the carcinogenesis of GCC in high-risk area might be related to chronic inflammation similar to the NGCC.

Previous studies suggested that TLR4 expression might be the link between *H*. *pylori* infection and cancer [31, 37, 58], and this expression pattern is not significantly changed after the eradication of bacteria [59]. In the present study, TLR4 expression was evaluated in a cohort of gastric cardia tissues. To our knowledge, our study is the first to evaluate TLR4 protein expression from normal mucosa to different degree of inflammation and carcinoma restricted to gastric cardia tissue. TLR4 expression gradually increased from normal mucosa to gastric cardia inflammation and carcinoma, thereby providing pathological evidence that TLR4 expression is involved in GCC inflammation and carcinogenesis. Similar to other studies, our study showed that normal gastric cardia cancer has a very low expression of TLR4 [58, 60]. TLR4 expression was greatly increased during chronic inflammation, and there was no significant difference between mild inflammation and severe inflammation, suggesting changes in innate immune activation between normal and mild inflammation. As we have seen, GCC has the highest and diffuse TLR4 expression. TLR4 expression was not only in the cytoplasm but also in the nucleus. It was speculated that at this phase, the presence of infection was not absolutely necessary for epithelial stimulation [58]. Though we did not detect TLR4 expression in EC tissue, previous studies showed TLR4 appeared important to the pathogenesis of esophageal squamous cell carcinoma [61]. Confirming the potential role of TLR4 in the progression of gastric lesions, some studies associated TLR4 polymorphisms with the risk of gastric cancer [34, 62–64]. TLR4+896A>G polymorphism was reported as a risk factor for NGCC and its precursors. In contrast, prevalence of TLR4+896G was not significantly increased in GCC [34]. Considering that GCC has different characteristics from NGCC, more molecular and functional studies about TLR4 in GCC are necessary, and distinguishing GCC from NGCC is encouraged.

In summary, this study showed that GCC carcinomas are biologically different from EC carcinomas in the Chaoshan high-risk area in China, although they share genetic risk factors and similar geographic aggregation. GCC in this high-risk area displays different characteristics from those of GEJ carcinomas in developed countries as well. We detected TLR4 expression in gastric cardia epithelial cells and demonstrate a progressive increase in TLR4 expression from normal gastric cardia tissue, gastric cardia inflammation, and GCC, which suggests that TLR4 plays a role in GCC carcinogenesis.

## 4. Methods

### 4.1. Study Group

All surgical pathology reports with a final diagnosis of EC and GCC were collected from the Tumor Hospital and the First Affiliated Hospital of Shantou University Medical College in China. Not all patients underwent radiotherapy and chemotreatment postoperatively. All cases were divided into EC and GCC groups based on the location of the tumor epicentre. Inclusion criteria were (1) surgical resection of tumors with lymph node dissection and (2) for GCC, the center of cancer within 2 cm of the GEJ on the gastric side. The GEJ was as defined by the WHO [46]. Most of the GCC tumors belonged to the AEG type II according to the Siewert classification [65]. Surgical details were collected principally from surgical notes and pathology findings of the resection specimen. GCCs were staged by the gastric TNM system, and ECs were staged by the esophageal TNM system, according to the American Joint Committee on Cancer Staging Manual [66]. Patients were followed for survival status by telephone or personal interview with the patient or family members. Patient consent for surgery and follow-up visit was obtained in all cases before surgical resection was performed. Informed consent was obtained from all patients. The methods were carried out in accordance with the approved guidelines. The Medical Ethics Committee of Shantou University Medical College approved the study protocol.

### 4.2. DNA Extraction and Amplification for Detecting *H*. *pylori* Infection

A total of 127 gastrointestinal mucosal tissue samples were collected from 83 patients with GCC and 44 with EC in the Tumor Hospital of Shantou University Medical College in China. Tissue DNA was extracted using a commercially available kit (Pure Link Genomic DNA Mini Kit, Invitrogen) according to the manufacturer's instructions [67]. The primers used were derived from the internal 300 bp fragment of the CagA as described [68]. The primer sequences used to detect CagA were 5′-ACCCTAGTCGGTAA TGGG-3′ and 5′-GCA AT TT TGT TAATCCGG TC-3′. These yielded a DNA fragment of 300 base pairs. A reaction mixture contained 3 *μ*L extracted DNA, 4 *μ*L primer, 3 *μ*L of 10x PCR buffer, 0.3 *μ*L AmpliTaq DNA polymerase, and 3 *μ*L dNTP. The amplification cycle consisted of an initial denaturation at 94°C for 3 min, followed by 34 cycles at 94°C for 30 s, 50°C for 45 s, 72°C for 1 min, and a final extension at 72°C for 10 min. PCR products were analyzed by electrophoresis on a 2% agarose gel stained with ethidium bromide.

### 4.3. Immunohistochemistry

Immunohistochemical (IHC) staining involved the Envision Labeled Peroxidase System (Dako, Carpinteria, CA). Paraffin-embedded samples were sectioned at 4 *μ*m. Each sample was deparaffinized in xylene, rehydrated in a graded ethanol series, then preincubated with 3% hydrogen peroxide for 10 min. Antigen retrieval was performed by microwave heating. Following incubation in 10 mmol/L citrate buffer for 20 min, sections were incubated with primary antibody for TLR4 (rabbit, 1 : 100, Proteintech) at 4°C overnight, then horseradish peroxidase-conjugated goat antirabbit IgG antibody at 37°C for 30 min, and counterstained with haematoxylin. Images were captured under a Leica IM50 microscope (Imagic Bildverarbeitung AG, Wetzlar, Germany).

IHC slides were evaluated by two experienced pathologists in a blinded manner. Staining intensity score (0–3) was considered according to a subjective evaluation of the intensity of marked cells (0: no immunostaining; 1: weak positive staining; 2: moderate positive staining; 3: strong positive staining). The overall staining intensity (0–3) was multiplied by the proportion of positive cells (0–100%), and all values were added to generate a final score ranging from 0 to 300 [69].

### 4.4. Chronic Inflammation Grading

Inflammation in the gastric cardia tissue was graded as normal, mild, and severe according to the updated Sydney System [70]. The normal gastric mucosa contains only individual (0–5) scattered inflammatory cells in the lamina propria. Mild inflammation contains 5 to 30 inflammatory cells in the lamina propria per high-power (×40 objective) microscopic field or between the foveolae. More than 30 inflammatory cells per high-power field was considered severe inflammation.

### 4.5. Statistical Analysis

Differences in frequencies between the two groups were analyzed by using chi-square or Fisher's exact test, as appropriate. Survival was estimated by the Kaplan-Meier method with a log-rank test. Cox regression analysis was used to identify risk factors for overall survival. *t*-test for trend was used to evaluate the trend in increase or decrease in expression. All statistical analyses involved the use of SPSS v16 (SPSS, Chicago, IL, USA). *P* < 0.05 was considered statistically significant.

## Figures and Tables

**Table 1:** Clinical and gross features of tumor patients with esophageal cancer (EC) and gastric cardia cancer (GCC).

Features	EC *n* = 84	GCC *n* = 99	*P*
Male : female ratio	63 : 21	84 : 15	0.095
Age, years, mean ± SD	57.14 ± 10.28	62.7 ± 7.79	0.003
Size, cm, mean ± SD	4.98 ± 1.51	5.92 ± 2.22	0.001
Epicentre location, *n* (%)			
Upper thoracic part	9 (10.71)	—	
Middle thoracic part	66 (78.57)	—	
Lower thoracic part	9 (10.71)	—	
Gastric cardia	—	99	

**Table 2:** Comparison of histopathology and pathological staging.

Microscopic features	EC *n* = 84	GCC *n* = 99	*P*
*Tumor differentiation*			<0.001
Well	29 (34.5)	5 (5.1)	
Moderate	50 (59.5)	49 (49.5)	
Poor	5 (6.0)	45 (45.5)	
*Histology type*			
Tubular adenocarcinoma	0 (0)	75 (75.8)	<0.001
Mucinous carcinoma	0 (0)	16 (16.2)	
Small-cell undifferentiated carcinoma	0 (0)	2 (2.0)	
Squamous cell carcinoma	84 (100)	1 (1.0)	
Adenosquamous	0 (0)	4 (4.0)	
Neuroendocrine carcinoma	0 (0)	1 (1.0)	
*Lymph node metastasis*	42 (50)	72 (72.7)	0.002
*Serosal invasion*	67 (70.8)	92 (92.9)	0.009
*TNM stage*			
0	0 (0)	0 (0)	<0.001
1	0 (0)	5 (5.1)	
2	40 (47.62)	10 (10.1)	
3	42 (50)	84 (84.8)	
4	2 (2.4)	0 (0)	

Data are *n* (%).

**Table 3:** *Helicobacter pylori* infection.

*H*. *pylori* infection	EC *n* = 44	GCC *n* = 83	*P*
Positive	19 (43.18)	40 (48.19)	0.59
Negative	25 (56.81)	43 (51.81)	

Data are *n* (%).

**Table 4:** Immunohistochemical evaluation of TLR4 expression.

	Normal *n* = 11	Mild carditis *n* = 44	Severe carditis *n* = 43	Carcinoma *n* = 103
TLR4	12.72 ± 15.71	28.63 ± 21.65^∗^	25.58 ± 22.23^∗^	63.67 ± 39.61^∗#^

TLR4: Toll-like receptor 4. Data are mean ± SD; ^∗^*P* < 0.05 versus normal mucosa; ^#^*P* < 0.05 versus mild or severe inflammation.
